# Frequency of cannabis use and symptoms of anxiety and depression: a cross-sectional analysis of the Colorado cannabis users health cohort

**DOI:** 10.1186/s42238-025-00327-2

**Published:** 2025-10-17

**Authors:** Christine M. Steeger, Poojashree Tandukar, Karin F. Hoth, Mark Aloia, Fred Wamboldt, Peter Castaldi, Sunita Sharma, Nancy Lorenzon, Laura E. Crotty Alexander, Jost Klawitter, Cristina Sempio, Gregory L. Kinney, Meghan D. Althoff, Gina R. Kruse, Russell P. Bowler

**Affiliations:** 1 University of Colorado Boulder, Boulder, Colorado USA; 2 Colorado State University Pueblo, Aurora, Colorado USA; 3 University of Iowa, Iowa Iowa City, USA; 4 National Jewish Health, Denver, Colorado USA; 5 Channing Laboratory of Network Medicine, Harvard University, Cambridge, Massachusetts USA; 6 University of Denver, Denver, Colorado USA; 7 University of California San Diego and VA San Diego Healthcare System, San Diego, California USA; 8 University of Colorado Denver, Denver, Colorado USA; 9 Cleveland Clinic, Cleveland, Ohio USA

**Keywords:** Cannabis use, Anxiety, Depression, Urinary cannabinoids, Adults

## Abstract

**Background:**

Cannabis is commonly used as a self-prescribed treatment for anxiety and depression, but few studies have evaluated these associations using both validated mental health scales and biological cannabinoid markers. This study aimed to test associations between cannabis use frequency and symptoms of anxiety and depression, and to examine whether frequent cannabis users with high symptom scores were less likely to use FDA-approved medications.

**Methods:**

This is a secondary analysis of a cross-sectional study on sleep and cannabis use, including 195 participants who completed the Hospital Anxiety and Depression Scale (HADS), Beck Anxiety Inventory (BAI), Beck Depression Inventory-II (BDI-II), and self-reported cannabis use. Urinary tetrahydrocannabinol (THC) metabolites validated recent cannabis exposure. Regression models adjusted for demographic and clinical variables.

**Results:**

Frequent cannabis use (≥ 15 uses in the past 30 days) vs. infrequent use (14 or fewer uses in the past 30 days) was associated with higher likelihood of anxiety, AOR = 1.06 (95% CI 1.01, 1.12), *p* < 0.01 for the BAI and AOR = 1.05 (95% CI 1.01, 1.09), *p* < 0.05 for the HADS-A. However, frequency of cannabis use was not associated with depression for either the HADS-D, AOR = 0.98 (95% CI 0.94, 1.05) or BDI-II, AOR = 0.98 (95% CI 0.92, 1.04), *ps* > 0.05. Use of FDA-approved anxiolytic or antidepressant medications did not significantly differ by non-use, infrequent, and frequent cannabis use groups (20% vs. 18.2% vs. 21.1% for anxiolytic-hypnotics and 14% vs. 9.1% vs. 11.4% for antidepressants), and urinary cannabinoid levels were not associated with symptom severity, all *ps* > 0.05.

**Conclusions:**

Elevated anxiety was common among frequent cannabis users, yet use of FDA-approved medications was infrequent in this group despite increased symptom burden. These results suggest that some individuals may turn to cannabis to manage their symptoms instead of using evidence-based treatments. Clinicians should consider the possibility that patients might substitute cannabis for prescription medications, and routinely screen cannabis users for untreated anxiety. Randomized studies are needed to determine causal associations between anxiety symptoms and cannabis use, including potential interactions with FDA-approved pharmacotherapies. Such evidence will inform clinical recommendations and policy on cannabis use and mental health.

**Supplementary Information:**

The online version contains supplementary material available at 10.1186/s42238-025-00327-2.

## Background

Mild to moderate anxiety and depressive symptoms are common in adults, with annual prevalence of anxiety disorders affecting 19% and major depressive episode affecting 8% of adults in the U.S., respectively (NIMH, 2017). Δ⁹-tetrahydrocannabinol (THC) has long been recognized as having potential anxiolytic effects, possibly directly through cannabinoid receptors (CB1R) or indirectly through benzodiazepine receptors (Berrendero and Maldonado 2002; García-Gutiérrez and Manzanares 2010; Witkin et al. 2005). Though evidence is mixed, recent reviews and meta-analyses generally show positive associations between greater cannabis use and higher frequencies of mental health conditions, with chronic cannabis users reporting the highest levels of mood and anxiety disorders, and psychosis symptomology (Martinotti and Di Forti 2025; Onaemo et al. 2021; Polkosnik et al. 2021; Sorkhou et al. 2021, 2024). Some studies show a dose-dependent relationship between cannabis use and mental health symptoms, with low doses of Δ⁹-THC producing anxiolytic effects and high doses producing anxiogenic responses (Petrilli et al. 2022; Sorkhou et al. 2021; Turna et al. 2017). Further, in the current cannabis legalization environment, THC potency has increased substantially over the past two decades (e.g., average ranges of 20% to above 90% THC, depending on the cannabis form and U.S. state dispensary regulations) (Chandra et al. 2019; Orens 2018). Although THC can show anxiolytic effects at low doses in laboratory settings, more research is needed to understand its associations with mood and anxiety symptoms at the higher concentrations now common in cannabis products.

One of the major side effects of anxiety and depressive symptoms is disturbed sleep, particularly insomnia (Staner 2003). Insomnia and difficulty sleeping are common reasons for cannabis use, as shown by decreases in over-the-counter sleep medication after introduction of dispensaries (Doremus et al. 2019). Studies have documented subjective improvements in sleep with cannabis use, however research to date has not observed improvements in sleep architecture among those using and not using cannabis, including our own work (Althoff et al. 2024; Lavender et al. 2022; Pacek et al. 2017; Velzeboer et al. 2022). The relationship between anxiety and depression and sleep disturbance may be explained by chronic stress leading to activation of the corticosteroid axis, resulting in chronic alertness (Hirotsu et al. 2015). Population studies suggest that anxiety disorders are present in roughly one quarter to one third of those with insomnia (Breslau et al. 1996). Further complicating the sleep-mental health disorder interactions are the adverse effects of cannabis withdrawal on mood, which may potentiate continued cannabis use (Bonnet and Preuss 2017). While legalization of medical and recreational cannabis has become more common, there are few studies examining the prevalence of anxiety and depression in those taking cannabis for sleep.

Cannabis is still a Schedule I drug in the U.S. (i.e., cannot be prescribed by physicians using a Drug Enforcement Administration [DEA] license), though it might become Schedule III and is more likely to be used outside of the medical establishment. Patients who use cannabis to treat medical disorders are more likely to seek homeopathic solutions to their medical problems and forsake U.S. Food and Drug Administration (FDA) approved medications (Wallis et al. 2022). Moreover, there is evidence that the legalization of cannabis has resulted in a 10–15% reduction in prescriptions for anxiolytic and antidepressant medications in states that have legalized cannabis (Bradford and Bradford 2016), despite the lack of clear evidence that recreational doses of THC have a beneficial impact on anxiety and depressive symptoms.

### Purpose of study

While existing studies have linked greater self-reported cannabis use frequency to more severe mental health symptoms (e.g., Leadbeater et al. 2019; Rup et al. 2021; Steeger et al. 2021), few have incorporated biological measures of THC to validate cannabis exposure or examined use of FDA-approved pharmacotherapy among individuals with elevated anxiety and depressive symptoms. To our knowledge, this is one of the first community-based studies to integrate multiple validated mental health scales, urinary THC biomarkers, and prescription medication data. This approach aims to clarify anxiety and depressive symptom burden among individuals who frequently self-administer cannabis, and how this cannabis use relates to use of pharmacotherapy for anxiety or depression. The findings will offer important insight into symptom burden and self-medication behavior, informing efforts to improve screening and treatment for adult cannabis users.

We conducted a secondary analysis of anxiety and depressive symptoms in a cohort of cannabis users and comparison participant non-users (controls) who were recruited for a study examining the impact of cannabis use on sleep. We hypothesized that participants who self-dosed cannabis frequently, as measured by self-report and urinary cannabinoids, will have more symptoms of anxiety and depression than those who infrequently self-dosed cannabis or cannabis users who have not used in the past 30 days. We further hypothesized that frequent cannabis users with high anxiety and depression scores would infrequently use traditional FDA-approved anxiolytic and antidepressant medications such as benzodiazepines, selective serotonin reuptake inhibitors (SSRIs), serotonin and norepinephrine inhibitors (SNRIs), tricyclic antidepressants (TCAs), monoamine oxidase inhibitors (MAOIs), etc.

## Methods

### Participants and procedures

The National Jewish Health Institutional Review Board approved this research. All participants consented to the study and were adults (aged 21–80 years) with less than 30 pack-year tobacco smoking history and were willing to complete a Home Sleep Test (HST). The age range was selected to encompass a broad adult demographic for comprehensive assessment. The exclusion of individuals with 30 pack-year or more of tobacco smoking history aimed to minimize the confounding effects of significant pulmonary comorbidities on sleep and other physiological measures relevant to the broader study aims, as this is a common criterion in respiratory and sleep research. Participants were recruited through local dispensaries, research bulletin boards, and word of mouth with flyers that included keywords such as cannabis and sleep.

Cases were defined as those who typically used cannabis > 3 days per week for at least one year and were further subdivided into infrequent (< 15 days in the past month) versus frequent (≥ 15 days in the past month). Further details on frequency cutoffs are described below in the measures section. Controls (non-cannabis users) were recruited from similar sources who had not used cannabis in the past 30 days.

**Fig. 1:**
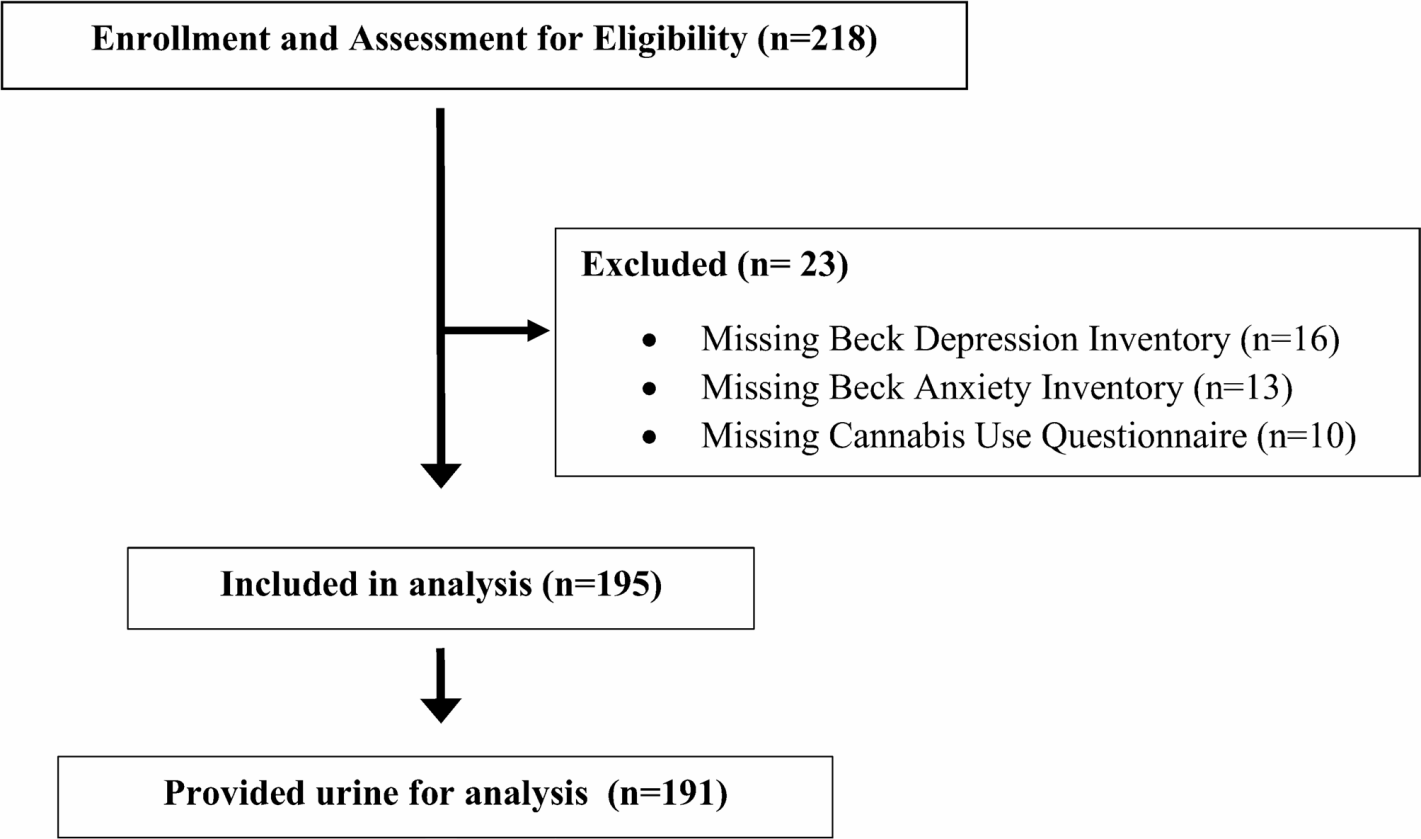
Flow diagram showing the number of participants enrolled and included in analyses

Participants enrolled in the study completed two study visits and underwent a HST over an ~ 18-hour period, described elsewhere in a parent study (Althoff et al. 2024). First, a baseline visit was conducted in the clinic in the evening. During this visit, participants completed questionnaires including demographic information and medical history, sleep quality, mental health, and cannabis use. They were also provided with sleep diaries and fitted for a HST (Althoff et al. 2024). Participants completed one night of diary tracking and HST recording and returned for the second and final follow-up visit the next morning to provide a blood and urine sample. A total of 218 participants were enrolled in the study from 2016 to 2019; 23 participants were excluded from the analysis due to incomplete or missing data for cannabis use or missing anxiety or depression measures (Fig. 1). Thus, 195 participants were included for analysis.

### Measures

Participants completed a medical history form regarding current and past medical conditions diagnosed by a medical provider (Supplemental Materials Table 1), substance use history, sleep quality (Pittsburgh Sleep Quality Index; PSQI; Buysse et al. 1989), and current medication use (Supplemental Materials Table 2).

#### Self-reported cannabis use

The cannabis use questionnaire included a detailed history of recent cannabis use. Participants were categorized into three groups based on the number of days they used cannabis in the past 30 days: non-users (0 days), infrequent users (1–14 days), and frequent users (15 or more days). The frequency of cannabis use cutoff scores used in this study divided the distribution of past 30-day use in our sample (see Supplemental Materials Table 3 for frequency of use information) and aligns with published thresholds indicating semi-frequent (10–19 days/month) and frequent (≥ 20 days/month) use (Caulkins et al., 2020; Lorenzetti et al., 2021; Sofis et al., 2020; Walsh et al., 2025). Thus, our 15 day cutoff captures use patterns of individuals who use cannabis on most days, reflecting elevated exposure.

#### Quantitation of cannabis in urine

Urine and blood assay methods are described elsewhere and included a panel of 11 total cannabinoids (Althoff et al. 2024; Klawitter et al. 2017). 11-nor-Δ9-tetrahydrocannabinol-9-carboxylic acid glucuronide (THC-COOH-gluc) (Althoff et al. 2024) is reported in the current study because most other cannabinoids, including cannabidiol (CBD), were below the limit of quantitation in most participants.

#### Self-reported anxiety and depressive symptoms

At enrollment, participants completed the 14-question Hospital Anxiety and Depression Scale (HADS), 21-question Beck Anxiety Inventory (BAI), and the 21-question Beck Depression Inventory-II (BDI-II). Moderate to severe (or clinically significant) anxiety and depression cutoffs for the HADS-Anxiety Subscale (HADS-A) and HADS-Depression Subscale (HADS-D) were both ≥ 8 (Bjelland et al. 2002; Zigmond and Snaith 1983). BDI-II cutoffs were: 0–13 minimal depression, 14–19 mild depression, 20–28 moderate depression, and 29–63 severe depression (Beck et al. 1996). BAI cutoffs were: 0–7 minimal anxiety, 8–15 mild anxiety, 16–25 moderate anxiety, and 26–63 severe anxiety (Julian 2011).

### Statistical analysis

All analyses were performed in SAS 9.4 or R 3.6.1. ANOVA was used to assess differences in demographic variables across the three cannabis use groups. Proportions were compared using NPAR1WAY Chi-square test. Multivariable logistic regression was used to examine the associations between the frequency of cannabis use (days of cannabis use per month, continuous variable) and elevated anxiety and depressive symptoms (HADS-A ≥ 8, BAI ≥ 16, HADS-D ≥ 8 and BDI-II ≥ 20). Regression analyses adjusted for age (years), sex (male/female), education (bachelor’s or more/less than bachelor’s), sleep quality (PSQI score), and anxiolytic-hypnotic use or antidepressant use (any use/no use). Linear regression was used to assess the relationship between HADS-A, BAI, HADS-D, and BDI-II scores and urinary cannabinoids. Variance inflation factors (VIF) were checked for model multicollinearity, and values were not elevated (VIF > 5) (see Supplemental Materials Table 4).

## Results

### Participant characteristics and cannabis use group comparisons

Participant demographic and substance use characteristics are shown in Table 1. Non-users of cannabis were more likely to report as female, have a bachelor’s degree or higher, earn more money, and have never used tobacco cigarettes, but were otherwise similar in age, race, employment status, and alcohol use compared to current (frequent or infrequent) cannabis users. Frequent cannabis users were more likely to be unmarried and used cigarettes compared to non-users or infrequent cannabis users.

**Table 1:** Description of participant characteristics

	Current (past 30 days) cannabis users			
	**Non-users** (*n* = 50)	**Infrequent** **Users** (< 15 days) (*n* = 22)	**Frequent users** (≥ 15 days) (*n* = 123)	*p*-value
**Age**	39.5 ± 17.2	41.9 ± 18.5	40.7 ± 14.2	NS
**Sex (Female)**	37 (74%)	11 (50%)	60 (50%)	0.0116
**Race (White)**	39 (78%)	17 (81%)	96 (79%)	NS
**Education Level (≥ Bachelor’s)**	42 (84%)	12 (57%)	46 (38%)	< 0.001
**Marital status**				0.0028
Married	21 (42%)	10 (45%)	24 (20%)	
Not Married	24 (48%)	6 (27%)	65 (53%)	
Widowed/ Separated/Divorced	5 (10%)	6 (27%)	33 (27%)	
**Employment (working now)**	36 (72%)	10 (52%)	70 (57%)	NS
**Family income**				0.0247
Less than US$35,000	12 (25%)	6 (32%)	50 (46%)	
US$35,000 - US$50,000	11 (23%)	3 (16%)	26 (24%)	
US$50,000 - US$75,000	12 (25%)	2 (10%)	12 (11%)	
US$75,000 - US$100,000	6 (12%)	2 (11%)	13 (12%)	
US$100,000 and above	7 (15%)	6 (31%)	8 (7%)	
**Tobacco Cigarette Use**				
Ever use	9 (18%)	6 (29%)	68 (56%)	< 0.0001
Current users	0 (0%)	1 (5%)	29 (24%)	0.0002
**Frequency of alcohol use**				
Never	4 (8.3)	4 (19.0)	19 (15.8)	NS
Monthly or less	9 (19%)	3 (14%)	24 (20%)	
2–4 times per month	22 (45%)	4 (19%)	33 (27%)	
2–3 times per week	10 (21%)	6 (29%)	32 (27%)	
≥ 4 times per week	3 (6%)	4 (19%)	12 (10%)	

*Note.* n (%) or mean ± standard deviation are shown. Not significant (NS) indicates *p* > 0.05

In current cannabis users, the reason for using cannabis at night was: to initiate sleep (78%) and/or to maintain sleep (48%). The major conditions that participants listed as the reason for taking cannabis at night were stress (57%), anxiety (52%), pain (46%), post-traumatic stress disorder (PTSD, 12%), and nightmares (9%). The prevalence of other medical conditions by cannabis frequency group were not significantly different among groups (Supplemental Materials Table 1).

### Anxiety and depression

Elevated anxiety was more frequent in cannabis users measured using both the HADS (HADS-A ≥ 8: 33% among frequent users, 18% among infrequent users, and 8% among non-users; *p* = 0.002) and the BAI instruments (BAI score ≥ 16: 30% among frequent users, 14% among infrequent users, and 0% among non-users, *p* < 0.001; Table 2). Despite much higher report of anxiety symptoms among frequent cannabis users, there was no significant difference in the use of FDA-approved anxiolytic-hypnotics between groups (21% among frequent users, 18% among infrequent users, and 20% among non-users), *p* > 0.05 (Table 2). Multivariable logistic regression models indicated that frequent cannabis use (days per month) was associated with higher likelihood of elevated anxiety, AOR = 1.06 (95% CI 1.01, 1.12), *p* < 0.01 for the BAI and AOR = 1.05 (95% CI 1.01, 1.09), *p* < 0.05 for the HADS-A (Table 3).

**Table 2:** Symptoms of anxiety and depression and use of commonly prescribed medication among participants

	Cannabis use in the past 30 days			
	**Non-users** 0 days (*n* = 50) n (%)	**Infrequent users** (< 15 days) (*n* = 22) n (%)	**Frequent users** (≥ 15 days) (*n* = 123) n (%)	Chi-square test *p*-value
**HADS anxiety score ≥ 8**	4 (8.0)	4 (18.2)	41 (33.3)	0.002
**HADS depression score ≥ 8**	2 (4.0)	1 (4.5)	13 (10.6)	NS
**Beck Anxiety Inventory**				
Minimal (0–7)	42 (84.0)	15 (68.2)	64 (52.0)	< 0.001
Mild (8–15)	8 (16.0)	4 (18.2)	22 (17.9)	
Moderate (16–25)	0 (0.0)	1 (4.5)	21 (17.1)	
Severe (26–63)	0 (0.0)	2 (9.1)	16 (13.0)	
**Beck Depression Inventory**				
Minimal (0–13)	48 (96.0)	20 (90.9)	92 (74.8)	0.011
Mild (14–19)	1 (2.0)	0 (0.0)	16 (13.0)	
Moderate (20–28)	1 (2.0)	1 (10.0)	10 (8.1)	
Severe (29–63)	0 (0.0)	0 (0.0)	5 (4.1)	
**Medication Usage**				
Anxiolytic-hypnotic	10 (20.0)	4 (18.2)	26 (21.1)	NS
Antidepressants	7 (14.0)	2 (9.1)	14 (11.4)	NS

*Note.* Total *N* = 195. HADS = Hospital Anxiety and Depression Scales. Not significant (NS) indicates *p* > 0.05

**Table 3:** Multivariable logistic regression models testing associations between cannabis use and elevated anxiety and depressive symptoms

	Anxiety		Depression	
	BAI ≥ 16	HADS-A ≥ 8	BDI-II ≥ 20	HADS-D ≥ 8
Age (per year)	0.97 (0.95, 1.00)	**0.95 (0.93**, 0.98)**	0.97 (0.93, 1.02)	1.00 (0.97, 1.04)
Gender (female)	1.58 (0.68, 3.71)	1.07 (0.49, 2.38)	1.42 (0.41, 4.94)	1.63 (0.49, 5.38)
Education Level (≥ Bachelor’s)	**0.69 (0.48**, 0.97)*	0.86 (0.62, 1.17)	0.83 (0.49, 1.40)	0.72 (0.45, 1.16)
PSQI	**1.18 (1.07**, 1.30)***	**1.24 (1.12**, 1.38)***	**1.37 (1.150**, 1.63)***	**1.17 (1.02**, 1.33)*
Cannabis Days (per month)	**1.06 (1.01**, 1.12)**	**1.05 (1.01**, 1.09)*	0.98 (0.92, 1.04)	0.99 (0.94, 1.05)

*Note*. Adjusted odds ratios with 95% CIs are shown. Significant odds ratios are bolded; *p < 0.05; **p < 0.01; ***p < 0.001; BAI = Beck Anxiety Inventory; HADS-Anxiety Subscale (HADS-A); BDI-II = Beck Depression Inventory; HADS-Depression Subscale (HADS-D); PSQI = Pittsburgh Sleep Quality Index

Clinically-elevated symptoms of depression (HADS-D score ≥ 8) were not significantly different across groups (11% among frequent users, 5% among infrequent users, and 4% among non-users), *p* > 0.05. However, using the BDI-II, frequent cannabis users were more likely to have moderate or severe symptoms of depression compared to infrequent users or never users (BDI-II ≥ 20: 12% vs. 10% vs. 2%, *p* = 0.011) (Table 2). Among frequent cannabis users, there were more participants with moderate or severe anxiety symptoms (30%) than moderate or severe depressive symptoms (12%). There was not a significant difference in use of antidepressants among cannabis use types (11% among frequent users, 9% among infrequent users, and 14% among non-users), *p* > 0.05 (Table 2). Finally, in multivariable regression models, the frequency of cannabis use was not associated with elevated depressive symptoms for either the BDI-II, AOR = 0.98 (95% CI 0.92, 1.04) or HADS-D, AOR = 0.99  (95% CI 0.94, 1.05), *ps* > 0.05 (Table 3).

### Urinary cannabinoids and anxiety and depressive symptoms

**Fig. 2:**
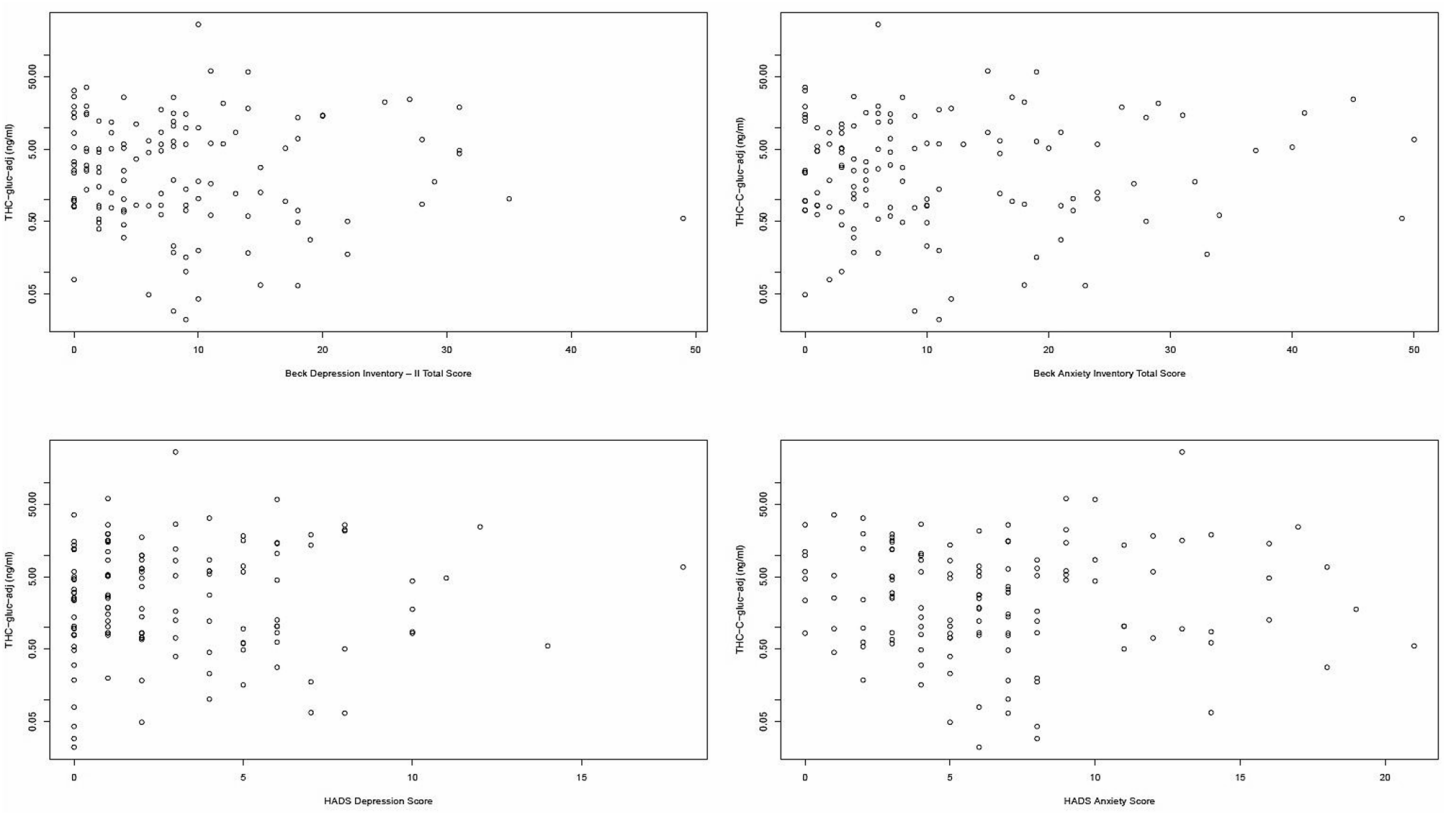
Relationship between urinary cannabinoids and depression and anxiety scores. Although there were wide ranges of urinary THC-glucuronide (THC-COOH-gluc) adjusted for urine/plasma creatinine and depression and anxiety scores, there was no statistically significant association between urinary cannabinoids and depression and anxiety scores

Although infrequent cannabis users had significantly less urinary cannabinoids compared to frequent users (1.46 ± 3.09 ng/ml versus 9.58 ± 26.2 ng/ml; *p* = 0.013; Supplemental Materials Fig. 1), there were no significant associations between urinary cannabinoids and the severity of anxiety symptoms or depressive symptoms, *ps* > 0.05 (Fig. 2).

## Discussion

This study found that frequent cannabis use in the past month was associated with elevated symptoms of anxiety but not depression, even after adjusting for key covariates. Though anxiety scores were higher among frequent cannabis users, there was no significant difference in the use of anxiolytic-hypnotic medications among frequent users, infrequent users, and non-users, which suggests that frequent users are not using FDA-approved medications as a primary method to treat their anxiety. Contrary to expectation, we also found no relationship between urinary THC-COOH-gluc (i.e., the highest detectable cannabinoid in this sample) and either elevated anxiety or depressive symptoms, suggesting that participants may not be fully self-titrating with cannabis to attenuate symptoms. It is also likely that at high levels of anxiety and depression, cannabis use is ineffective in fully controlling symptoms (Feingold et al. 2015). Although anxiety symptoms were higher in frequent cannabis users for both anxiety measures, there was a significant difference in BDI-II depression scores but not HADS-D scores. Additionally, the use of antidepressant medications was similar across groups.

Although the HADS-D did not indicate significant group differences in depression, the BDI-II found higher levels of moderate to severe depression for frequent cannabis users. This discrepancy may reflect differences in how the two depression measures assess symptoms. The BDI-II includes somatic symptoms (e.g., sleep disturbance, fatigue), which may be relevant to frequent users in this community-based sample, in addition to affective and cognitive symptoms.

Though it is difficult to speculate about the reasons for null findings, there are several possible explanations for why there could have been significant associations between cannabis use and anxiety symptoms but not depressive symptoms. Results may be due to differences in the acute self-medicating potential of cannabis for anxiety (particularly with CBD-dominant strains; Bidwell et al. 2024; Kosiba et al. 2019; Sharpe et al. 2020). There is also some evidence for a stronger neurobiological overlap between cannabinoids and anxiety-related circuits (e.g., CB1R) (Lutz et al. 2015) than depression pathways. Finally, in the current study, participants’ most prevalent reasons for cannabis use were for sleep, anxiety, stress, and pain reduction rather than depressive symptoms. Prospective studies and randomized controlled trials (RCTs) are needed to differentiate symptom-specific motivations and neurobiological pathways linking cannabis use and mental health (Hasbi et al. 2023).

Anxiety disorders are among the most prevalent mental health conditions worldwide (Kessler et al. 2010); yet nearly half of sufferers do not achieve complete relief with pharmacologic treatment (Bandelow et al. 2008). Endocannabinoids such as anandamide (AEA), metabolizing enzymes such as fatty acid amide hydrolase (FAAH), and CB1R have all been shown experimentally to play an important role in anxiety (Patel et al. 2017; Scherma et al. 2018). CB1R is found extensively throughout the brain in regions such as the amygdala, hippocampus, and prefrontal cortex and are likely to mediate most of the anxiolytic effects of cannabinoids such as THC, although there is still uncertainty about the role other cannabinoids (such as CBD) play in these pathways (Patel et al. 2017). Further evidence to support a role for cannabinoids in treating mental health includes a cross-over RCT of 10 participants in which the synthetic cannabinoid nabilone reduced PTSD-associated nightmares (Jetly et al. 2015). Additionally, a meta-analysis of 14 clinical studies of anxiety disorders (including six RCTs) showed a -1.85 standardized mean difference in subjects who received cannabinoids without significant adverse effect (Bahji et al. 2020). Thus, it is not surprising that cannabis is currently being used as a self-remedy by many participants.

The current study shows that frequent cannabis users had higher BAI and HADS-A scores than non-frequent users or non-users. This is consistent with recent reports indicating that individuals with anxiety disorders or depression who self-medicate with cannabis have a more severe course and worse symptoms (Bahorik et al. 2017; Beletsky et al. 2024; Wilkinson et al. 2015). In our study, frequent cannabis use was still associated with elevated anxiety symptoms after controlling for confounders. One explanation may be self-titration, a concept supported by studies showing that use of cannabinoids can have anxiolytic effects at low doses but may provoke anxiety at higher doses (Turna et al. 2017; Viveros et al. 2005). However, we did not collect longitudinal measurements on changes in anxiety after cannabis use and cannot comment on the impact of cannabis on anxiety symptoms. Although cessation of cannabis was not part of this study, the short half-life of cannabinoids combined with self-medication behavior may lead cannabis users to rely on cannabis for short-term relief, unaware that frequent use could perpetuate a long-term usage because of anxiety induced by withdrawal.

A major challenge for cannabis research is assessing dose. To our knowledge, our study is among the first to include quantitative urinary cannabinoid measurements to objectively assess the relationship between dose and severity of anxiety and depressive symptoms. While there was a strong association between self-reported frequency of dosing and urinary cannabinoids, there was no association between urinary cannabinoids and severity of depression. This result confirms the lack of association between self-reported frequency of use and severity of depressive symptoms and suggests accuracy in self-reported use data for the current sample. Interestingly, while using cannabis more days per month was associated with elevated anxiety, the urinary cannabinoid levels were not associated with severity of anxiety scores, suggesting that daily use may be more important than high doses of cannabis.

Our observation of a low prevalence of anxiolytics in participants who have elevated levels of anxiety supports the idea that participants may be self-treating with cannabis instead of using FDA-approved medications, though we cannot exclude barriers to seeking medical care for anxiety and depression in medication differences between groups. Differences between the Bradford and Bradford (2016) study and the current study were primarily that the authors included only Medicare patients, who are older, and represent only 21% of medical cannabis users in Colorado (CDPHE, 2020). Additionally, Bradford & Bradford did not account for the legalization of recreational cannabis use, which occurred in 2012 in Colorado. Furthermore, pain is by far the most reported therapeutic target of medical cannabis use and anxiety and depression are not listed as conditions for medical use in Colorado. The observed infrequent use of FDA-approved anxiolytic medications in participants with high anxiety scores who frequently use cannabis suggests potential for both medication substitution and self-management of symptoms.

The finding that frequent cannabis users with elevated anxiety and depression scores were not more likely to use FDA-approved treatments is consistent with prior evidence on self-medication and under-treatment in this population. Past research shows that individuals may substitute cannabis for anxiolytics or antidepressants (Corroon et al., 2017; Kvamme et al., 2021; Lucas & Walsh, 2017), and many people with mood or anxiety symptoms are undertreated (Lamoureux-Lamarche, Berbiche, & Vasiliadis, 2022; Weisberg et al., 2014). The current study adds to the cannabis use and mental health literature by demonstrating this pattern using both validated anxiety and depressive symptom scales and biomarker-confirmed cannabis use.

### Limitations and future directions

While this study is among the largest to investigate the relationship between anxiety and depression and biologic measurements of cannabinoids, there are several limitations. First, the study was cross-sectional, and we were not able investigate the relationship between cannabis use and mental health over time to determine whether frequent cannabis use causes future mood and anxiety symptoms, mood and anxiety symptoms lead to more frequent cannabis use, or whether associations are bidirectional. In an Australian cohort study of adolescents, daily cannabis use was associated with a higher prevalence of depression and anxiety six years later; however, anxiety or depression in adolescence were not associated with more frequent cannabis use over time (Patton et al. 2002). The Patton et al. study contrasts results from the current research, which only includes adults. Mixed findings in this area underscore the need for prospective research to investigate the direction of associations between cannabis use and mental health symptoms, and how associations may vary across adolescent, young adult, and adult age groups (Gobbi et al. 2019; Lowe et al. 2024; Sorkhou et al. 2021, 2024).

Second, while the strongest determinant of urinary cannabinoids was frequency of use and not type of use, we were not statistically powered to assess whether method of cannabis use (e.g., smoking, vaping, edibles, dabbing, etc.) was associated with severity of anxiety and depression. Additionally, this community-based sample recruited for a study on sleep and cannabis use had complex patterns of use, including various methods of use, products, and dosage particularly for daily users, which made it difficult to determine predominant cannabis use methods and products. Third, the current study did not systematically assess cannabis potency (% THC and CBD concentration) or quantity, which are important considerations when examining frequency of use and mental health symptoms given the range of cannabis strains and products currently available in the legal market (Prince and Conner 2019; Steeger et al. 2021). Though some recent reviews on cannabis potency and mental health show that THC is the primary cannabinoid responsible for the health effects of cannabis use (Petrilli et al. 2022), there is mixed evidence of the therapeutic role of CBD in reducing mental health symptoms (Khan et al. 2020). Well-designed clinical trials and longitudinal research are needed to further understand how CBD and other cannabinoids may moderate the effects of THC on anxiety and depressive symptoms (Bhuller et al. 2024; Bidwell et al. 2024; Khan et al. 2020).

Fourth, this study was not funded to quantitate nicotine in addition to THC. Future studies should examine whether tobacco smoking may be a confounding factor of associations among nicotine use, anxiety, and sleep disturbances. Fifth, this research was designed as an observational study of frequent users, primarily recruited from cannabis dispensaries, which can introduce selection bias including study participants who may not generalize to broader or clinical populations with diagnosed mental health conditions. Medical cannabis vs. recreational cannabis use status was not available for this sample and should be measured in future studies given some evidence of different cannabis use patterns for medically- vs. recreationally-motivated cannabis user types, including different product preferences and cannabinoid profiles (Sznitman 2017; Turna et al. 2020). However, in the current study, participants had zero to low detectable levels of CBD in biological specimens, which suggests that regardless of medical or recreational user status, this sample of cannabis users largely used THC-dominant products. Sixth, participants did not endorse depression as a common reason for cannabis use. Thus, the lack of association between frequent cannabis use and depression may be sample-specific. Finally, while we did not specifically recruit those with sleep disorders, the primary study objective of understanding cannabis and sleep using a HST may have led to increased participation among those with sleep problems, impacting generalizability of our findings.

## Conclusions

We found that elevated anxiety is common in frequent cannabis users, but those with elevated anxiety were not more likely to be taking FDA-approved anxiolytic medications. These findings support the concept that cannabis users may be self-treating their anxiety with cannabis. Our findings suggest that anxiety symptom screening among adults who use cannabis may present opportunities to connect individuals with FDA-approved anxiolytic/hypnotic treatment. There is also a clear need for prospective studies and randomized trials to determine causal associations between cannabis use and anxiety or depressive symptoms, including any interactions between cannabis use and anxiolytics or antidepressants on mental health symptoms.

## Supplementary Information

Below is the link to the electronic supplementary material.

Supplementary Material 1

## Data Availability

Data are available on request from the author RB.

## References

[CR1] Althoff MD, Kinney GL, Aloia MS, Sempio C, Klawitter J, Bowler RP. (2024). The impact of cannabis use proximal to sleep and cannabinoid metabolites on sleep architecture. Journal of Clinical Sleep Medicine, jcsm. 11212. 10.5664/jcsm.11212 PMC11446118 38804689

[CR2] Bahji A, Meyyappan AC, Hawken ER. Efficacy and acceptability of cannabinoids for anxiety disorders in adults: A systematic review & meta-analysis. J Psychiatr Res. 2020;129:257–64. 10.1016/j.jpsychires.2020.07.030. 32827809 10.1016/j.jpsychires.2020.07.030

[CR3] Bahorik AL, Leibowitz A, Sterling SA, Travis A, Weisner C, Satre DD. Patterns of marijuana use among psychiatry patients with depression and its impact on recovery. J Affect Disord. 2017;213:168–71. 10.1016/j.jad.2017.02.016. 28242498 10.1016/j.jad.2017.02.016 PMC5407687

[CR4] Bandelow B, Zohar J, Hollander E, Kasper S, Moller HJ, WFSBP Task Force on Treatment Guidelines for Anxiety, Post-Traumatic Stress O-C, Zohar D, Hollander J, Kasper E, Moller S, Bandelow HJ, Allgulander B, Ayuso-Gutierrez C, Baldwin J, Buenvicius DS, Cassano R, Fineberg G, Gabriels N, Vega L. J. World Federation of Societies of Biological Psychiatry (WFSBP) guidelines for the pharmacological treatment of anxiety, obsessive-compulsive and post-traumatic stress disorders - first revision. World J Biol Psychiatry. 2008;9(4):248–312. 10.1080/15622970802465807 10.1080/15622970802465807 18949648

[CR5] Beck AT, Steer RA, Brown GK. BDI-II, Beck depression inventory: manual. 2nd ed. Psychological Corp.; 1996.

[CR6] Beletsky A, Liu C, Lochte B, Samuel N, Grant I. Cannabis and anxiety: A critical review. Med Cannabis Cannabinoids. 2024;7(1):19–30. 38406383 10.1159/000534855 PMC10890807

[CR7] Berrendero F, Maldonado R. Involvement of the opioid system in the anxiolytic-like effects induced by Delta(9)-tetrahydrocannabinol. Psychopharmacology. 2002;163(1):111–7. 10.1007/s00213-002-1144-9. 12185408 10.1007/s00213-002-1144-9

[CR8] Bhuller R, Schlage WK, Hoeng J. Review of the current ongoing clinical trials exploring the possible anti-anxiety effects of Cannabidiol. J Cannabis Res. 2024;6(1):1–23. 39394179 10.1186/s42238-024-00250-y PMC11481052

[CR9] Bidwell LC, Martin-Willett R, Skrzynski C, Lisano J, Ortiz Torres M, Giordano G, Hutchison KE, Bryan AD. Acute and extended anxiolytic effects of Cannabidiol in cannabis flower: A quasi-experimental ad libitum use study. Cannabis Cannabinoid Res. 2024;9(4):1015–27. 38252547 10.1089/can.2023.0187 PMC11392455

[CR10] Bjelland I, Dahl AA, Haug TT, Neckelmann D. The validity of the hospital anxiety and depression scale: an updated literature review. J Psychosom Res. 2002;52(2):69–77. 11832252 10.1016/s0022-3999(01)00296-3

[CR11] Bonnet U, Preuss UW. The cannabis withdrawal syndrome: current insights. Subst Abuse Rehabil. 2017;8:9–37. 10.2147/SAR.S109576. 28490916 10.2147/SAR.S109576 PMC5414724

[CR12] Bradford AC, Bradford WD. (2016). Medical Marijuana Laws Reduce Prescription Medication Use In Medicare Part D. https://www.healthaffairs.org/doi/10.1377/hlthaff.2015.1661 10.1377/hlthaff.2015.1661 27385238

[CR13] Breslau N, Roth T, Rosenthal L, Andreski P. Sleep disturbance and psychiatric disorders: a longitudinal epidemiological study of young adults. Biol Psychiatry. 1996;39(6):411–8. 10.1016/0006-3223(95)00188-3. 8679786 10.1016/0006-3223(95)00188-3

[CR14] Buysse DJ, Reynolds III, Monk CF, Berman TH, S. R., Kupfer DJ. The Pittsburgh sleep quality index: a new instrument for psychiatric practice and research. Psychiatry Res. 1989;28(2):193–213. 2748771 10.1016/0165-1781(89)90047-4

[CR60] Caulkins JP, Pardo B, Kilmer B. Intensity of cannabis use: Findings from three online surveys. Int J Drug Policy. 2020;79:102740. 10.1016/j.drugpo.2020.102740 32334336

[CR15] Chandra S, Radwan MM, Majumdar CG, Church JC, Freeman TP, ElSohly MA. New trends in cannabis potency in USA and Europe during the last decade (2008–2017). Eur Arch Psychiatry Clin NeuroSci. 2019;269:5–15. 30671616 10.1007/s00406-019-00983-5

[CR16] Colorado Department of Public Health and Environment (CDPHE). (2020). Medical marijuana statistics and data. Retrieved 8 Oct 2024, from https://cdphe.colorado.gov/medical-marijuana-registry-data

[CR64] Corroon Jr JM, Mischley LK, Sexton M. Cannabis as a substitute for prescription drugs–a cross-sectional study. J Pain Res. 2017:989–998. 10.2147/JPR.S134330 PMC5422566 28496355

[CR17] Doremus JM, Stith SS, Vigil JM. Using recreational cannabis to treat insomnia: evidence from over-the-counter sleep aid sales in Colorado. Complement Ther Med. 2019;47:102207. 31779999 10.1016/j.ctim.2019.102207

[CR18] Feingold D, Weiser M, Rehm J, Lev-Ran S. The association between cannabis use and mood disorders: A longitudinal study. J Affect Disord. 2015;172:211–8. 25451420 10.1016/j.jad.2014.10.006

[CR19] García-Gutiérrez MS, Manzanares J. The cannabinoid CB1 receptor is involved in the anxiolytic, sedative and amnesic actions of benzodiazepines. J Psychopharmacol. 2010;24(5):757–65. 19825899 10.1177/0269881109106910

[CR20] Gobbi G, Atkin T, Zytynski T, Wang S, Askari S, Boruff J, Ware M, Marmorstein N, Cipriani A, Dendukuri N. Association of cannabis use in adolescence and risk of depression, anxiety, and suicidality in young adulthood: a systematic review and meta-analysis. JAMA Psychiatry. 2019;76(4):426–34. 30758486 10.1001/jamapsychiatry.2018.4500 PMC6450286

[CR21] Hasbi A, Madras BK, George SR. Endocannabinoid system and exogenous cannabinoids in depression and anxiety: A review. Brain Sci. 2023;13(2):325. 36831868 10.3390/brainsci13020325 PMC9953886

[CR23] Hirotsu C, Tufik S, Andersen ML. Interactions between sleep, stress, and metabolism: from physiological to pathological conditions. Sleep Sci. 2015;8(3):143–52. 10.1016/j.slsci.2015.09.002. 26779321 10.1016/j.slsci.2015.09.002 PMC4688585

[CR24] Jetly R, Heber A, Fraser G, Boisvert D. The efficacy of nabilone, a synthetic cannabinoid, in the treatment of PTSD-associated nightmares: A preliminary randomized, double-blind, placebo-controlled cross-over design study. Psychoneuroendocrinology. 2015;51:585–8. 10.1016/j.psyneuen.2014.11.002. 25467221 10.1016/j.psyneuen.2014.11.002

[CR25] Julian LJ. Measures of anxiety: State-Trait anxiety inventory (STAI), Beck anxiety inventory (BAI), and hospital anxiety and depression Scale-Anxiety (HADS-A). Arthritis Care Res (Hoboken). 2011;63(Suppl 11):S467–472. 10.1002/acr.20561. 22588767 10.1002/acr.20561 PMC3879951

[CR26] Kessler RC, Ruscio AM, Shear K, Wittchen HU. Epidemiology of anxiety disorders. Curr Top Behav Neurosci. 2010;2:21–35. https://www.ncbi.nlm.nih.gov/pubmed/21309104. 21309104

[CR27] Khan R, Naveed S, Mian N, Fida A, Raafey MA, Aedma KK. The therapeutic role of Cannabidiol in mental health: A systematic review. J Cannabis Res. 2020;2:1–21. 10.1186/s42238-019-0012-y PMC7819291 33526132

[CR28] Klawitter J, Sempio C, Morlein S, De Bloois E, Klepacki J, Henthorn T, Leehey MA, Hoffenberg EJ, Knupp K, Wang GS, Hopfer C, Kinney G, Bowler R, Foreman N, Galinkin J, Christians U, Klawitter J. An atmospheric pressure chemical ionization MS/MS assay using online extraction for the analysis of 11 cannabinoids and metabolites in human plasma and urine. Ther Drug Monit. 2017;39(5):556–64. 10.1097/FTD.0000000000000427. 28640062 10.1097/FTD.0000000000000427 PMC5600652

[CR29] Kosiba JD, Maisto SA, Ditre JW. Patient-reported use of medical cannabis for pain, anxiety, and depression symptoms: systematic review and meta-analysis. Soc Sci Med. 2019;233:181–92. 31207470 10.1016/j.socscimed.2019.06.005

[CR65] Kvamme SL, Pedersen MM, Rømer Thomsen K, Thylstrup B. Exploring the use of cannabis as a substitute for prescription drugs in a convenience sample. Harm Reduct J. 2021;18(1):72. 10.1186/s12954-021-00520-5 PMC8272272 34246279

[CR68] Lamoureux-Lamarche C, Berbiche D, Vasiliadis HM. Health care system and patient costs associated with receipt of minimally adequate treatment for depression and anxiety disorders in older adults. BMC psychiatry. 2022;22(1):175. 10.1186/s12888-022-03759-9 PMC8908583 35272650

[CR30] Lavender I, McGregor IS, Suraev A, Grunstein RR, Hoyos CM. Cannabinoids, insomnia, and other sleep disorders. Chest. 2022;162(2):452–65. 35537535 10.1016/j.chest.2022.04.151

[CR31] Leadbeater BJ, Ames ME, Linden-Carmichael AN. Age‐varying effects of cannabis use frequency and disorder on symptoms of psychosis, depression and anxiety in adolescents and adults. Addiction. 2019;114(2):278–93. 30276906 10.1111/add.14459 PMC6519223

[CR61] Lorenzetti V, Hindocha C, Petrilli K, Griffiths P, Brown J, Castillo‐Carniglia Á, et al. The International Cannabis Toolkit (iCannToolkit): a multidisciplinary expert consensus on minimum standards for measuring cannabis use. Addiction. 2021;117(6):510–1517 10.1111/add.15702 PMC9298052 34590359

[CR32] Lowe DJ, Sorkhou M, George TP. Cannabis use in adolescents and anxiety symptoms and disorders: a systematic review and meta-analysis. Am J Drug Alcohol Abus. 2024;50(2):150–61. 10.1080/00952990.2023.2299922 38285048

[CR66] Lucas P, Walsh Z. Medical cannabis access, use, and substitution for prescription opioids and other substances: a survey of authorized medical cannabis patients. Int J Drug Policy. 2017;42:30–35. 10.1016/j.drugpo.2017.01.011 28189912

[CR33] Lutz B, Marsicano G, Maldonado R, Hillard CJ. The endocannabinoid system in guarding against fear, anxiety and stress. Nat Rev Neurosci. 2015;16(12):705–18. 26585799 10.1038/nrn4036 PMC5871913

[CR34] Martinotti G, Di Forti M. Cannabis use and psychosis: evidence and new clinical features of a new epidemic. Eur Neuropsychopharmacology: J Eur Coll Neuropsychopharmacol. 2025;91:45–6. 10.1016/j.euroneuro.2024.11.007 39612729

[CR35] National Institute of Mental Health (NIMH). (2017). Statistics. Retrieved 10 Dec 2024, from www.nimh.nih.gov/health/statistics

[CR36] Onaemo VN, Fawehinmi TO, D’Arcy C. Comorbid cannabis use disorder with major depression and generalized anxiety disorder: A systematic review with meta-analysis of nationally representative epidemiological surveys. J Affect Disord. 2021;281:467–75. 33360749 10.1016/j.jad.2020.12.043

[CR37] Orens AD. Marketing size and demand for marijuana in Colorado 2017, market update. Marijuana Policy Group; 2018.

[CR38] Pacek LR, Herrmann ES, Smith MT, Vandrey R. Sleep continuity, architecture and quality among treatment-seeking cannabis users: an in-home, unattended polysomnographic study. Exp Clin Psychopharmacol. 2017;25(4):295. 28782982 10.1037/pha0000126 PMC6309181

[CR39] Patel S, Hill MN, Cheer JF, Wotjak CT, Holmes A. The endocannabinoid system as a target for novel anxiolytic drugs. Neurosci Biobehav Rev. 2017;76(Pt A):56–66. 10.1016/j.neubiorev.2016.12.033. 28434588 10.1016/j.neubiorev.2016.12.033 PMC5407316

[CR40] Patton GC, Coffey C, Carlin JB, Degenhardt L, Lynskey M, Hall W. Cannabis use and mental health in young people: cohort study. BMJ. 2002;325(7374):1195–8. 10.1136/bmj.325.7374.1195. 12446533 10.1136/bmj.325.7374.1195 PMC135489

[CR41] Petrilli K, Ofori S, Hines L, Taylor G, Adams S, Freeman TP. Association of cannabis potency with mental ill health and addiction: A systematic review. Lancet Psychiatry. 2022;9(9):736–50. 35901795 10.1016/S2215-0366(22)00161-4

[CR42] Polkosnik GL, Sorkhou M, George TP. Effects of cannabis use on psychotic and mood symptoms: a systematic review. Can J Addict. 2021;12(3):10–21.

[CR43] Prince MA, Conner BT. Examining links between cannabis potency and mental and physical health outcomes. Behav Res Ther. 2019;115:111–20. 30497655 10.1016/j.brat.2018.11.008

[CR44] Rup J, Freeman TP, Perlman C, Hammond D. Cannabis and mental health: prevalence of use and modes of cannabis administration by mental health status. Addict Behav. 2021;121:106991. 34087766 10.1016/j.addbeh.2021.106991

[CR45] Scherma M, Masia P, Deidda M, Fratta W, Tanda G, Fadda P. New perspectives on the use of cannabis in the treatment of psychiatric disorders. Med (Basel). 2018;5(4). 10.3390/medicines5040107. 10.3390/medicines5040107 PMC6313625 30279403

[CR46] Sharpe L, Sinclair J, Kramer A, De Manincor M, Sarris J. Cannabis, a cause for anxiety? A critical appraisal of the anxiogenic and anxiolytic properties. J Translational Med. 2020;18(1):374. 10.1186/s12967-020-02518-2 PMC7531079 33008420

[CR62] Sofis MJ, Budney AJ, Stanger C, Knapp AA, Borodovsky JT. Greater delay discounting and cannabis coping motives are associated with more frequent cannabis use in a large sample of adult cannabis users. Drug Alcohol Depend. 2020;207:107820. 10.1016/j.drugalcdep.2019.107820 PMC7147078 31887604

[CR47] Sorkhou M, Bedder RH, George TP. The behavioral sequelae of cannabis use in healthy people: A systematic review. Front Psychiatry. 2021;12:630247. 33664685 10.3389/fpsyt.2021.630247 PMC7920961

[CR48] Sorkhou M, Dent EL, George TP. Cannabis use and mood disorders: A systematic review. Front Public Health. 2024;12:1346207. 38655516 10.3389/fpubh.2024.1346207 PMC11035759

[CR49] Staner L. Sleep and anxiety disorders. Dialogues Clin Neurosci. 2003;5(3):249–58. https://www.ncbi.nlm.nih.gov/pubmed/22033804. 22033804 10.31887/DCNS.2003.5.3/lstaner PMC3181635

[CR50] Steeger CM, Hitchcock LN, Bryan AD, Hutchison KE, Hill KG, Bidwell LC. Associations between self-reported cannabis use frequency, potency, and cannabis/health metrics. Int J Drug Policy. 2021;97:103278. 34062287 10.1016/j.drugpo.2021.103278 PMC8585676

[CR51] Sznitman SR. Do recreational cannabis users, unlicensed and licensed medical cannabis users form distinct groups? Int J Drug Policy. 2017;42:15–21. 28107687 10.1016/j.drugpo.2016.11.010

[CR52] Turna J, Balodis I, Munn C, Van Ameringen M, Busse J, MacKillop J. Overlapping patterns of recreational and medical cannabis use in a large community sample of cannabis users. Compr Psychiatr. 2020;102:152188. 10.1016/j.comppsych.2020.152188 32653594

[CR53] Turna J, Patterson B, Van Ameringen M. Is cannabis treatment for anxiety, mood, and related disorders ready for prime time? Depress Anxiety. 2017;34(11):1006–17. 10.1002/da.22664. 28636769 10.1002/da.22664

[CR54] Velzeboer R, Malas A, Boerkoel P, Cullen K, Hawkins M, Roesler J, Lai WW-K. Cannabis dosing and administration for sleep: A systematic review. Sleep. 2022;45(11):zsac218. 36107800 10.1093/sleep/zsac218

[CR55] Viveros MP, Marco EM, File SE. Endocannabinoid system and stress and anxiety responses. Pharmacol Biochem Behav. 2005;81(2):331–42. 10.1016/j.pbb.2005.01.029. 15927244 10.1016/j.pbb.2005.01.029

[CR56] Wallis D, Coatsworth JD, Mennis J, Riggs NR, Zaharakis N, Russell MA, Brown AR, Rayburn S, Radford A, Hale C, Mason MJ. Predicting Self-Medication with cannabis in young adults with hazardous cannabis use. Int J Environ Res Public Health. 2022;19(3). 10.3390/ijerph19031850. 10.3390/ijerph19031850 PMC8834899 35162872

[CR63] Walsh CA, Euler E, Do LA, Zheng A, Eckel SP, Harlow BL, et al. Cannabis use and sleep problems among young adults by mental health status: A prospective cohort study. Addiction. 2025;120(4):688–696. 10.1111/add.16705 PMC11908928 39476498

[CR67] Weisberg RB, Beard C, Moitra E, Dyck I, Keller MB. Adequacy of treatment received by primary care patients with anxiety disorders. Depression and Anxiety. 2014;31(5):443–450. 10.1002/da.22209 PMC4157338 24190762

[CR57] Wilkinson ST, Stefanovics E, Rosenheck RA. Marijuana use is associated with worse outcomes in symptom severity and violent behavior in patients with posttraumatic stress disorder. J Clin Psychiatry. 2015;76(9):1174–80. 10.4088/JCP.14m09475. 26455669 10.4088/JCP.14m09475 PMC6258013

[CR58] Witkin JM, Tzavara E, Nomikos G. A role for cannabinoid CB1 receptors in mood and anxiety disorders. Behav Pharmacol. 2005;16(5–6):315–31. 16148437 10.1097/00008877-200509000-00005

[CR59] Zigmond AS, Snaith RP. The hospital anxiety and depression scale. Acta Psychiatr Scand. 1983;67(6):361–70. 10.1111/j.1600-0447.1983.tb09716.x. 6880820 10.1111/j.1600-0447.1983.tb09716.x

