# Supplementary material for: Frequency of cannabis use and symptoms of anxiety and depression: a cross-sectional analysis of the Colorado cannabis users health cohort
Source: J Cannabis Res. 2025 Oct 17;7:78. doi: 10.1186/s42238-025-00327-2 (PMC12535088; doi:10.1186/s42238-025-00327-2)
Supplement: Supplementary file 1 — Supplementary Material 1 [file 42238_2025_327_MOESM1_ESM.docx]

**Supplemental Materials**

| Supplemental Table 1. Prevalence of medical conditions across different cannabis use frequencies in past 30 days | | | |
| --- | --- | --- | --- |
|  | Never User | 1-15 days | 16-30 days |
| Angina | 0% | 0% | 2% |
| Atrial Fibrillation | 0% | 4% | 2% |
| Congestive Heart Failure | 0% | 0% | 0% |
| Coronary Artery Disease | 0% | 4% | 0% |
| Diabetes | 4% | 4% | 3% |
| High Blood Pressure | 10% | 11% | 17% |
| High Cholesterol | 12% | 15% | 16% |
| Heart Attack | 0% | 0% | 1% |
| Blood Clots | 2% | 0% | 2% |
| Macular Degeneration | 0% | 4% | 0% |
| Peripheral Vascular Disease | 0% | 4% | 0% |
| Transient Ischemic Attack | 0% | 0% | 1% |
| Stroke | 0% | 0% | 0% |
| Cognitive (memory) Disorder | 0% | 4% | 4% |
| Anemia | 10% | 15% | 12% |
| GERD | 8% | 11% | 18% |
| HIV/AIDS | 0% | 0% | 0% |
| Kidney Disease | 2% | 0% | 2% |
| Rheumatoid Arthritis | 0% | 4% | 2% |
| Osteoarthritis | 2% | 11% | 6% |
| Osteoporosis | 6% | 0% | 2% |
| Hip Fractures | 0% | 0% | 1% |
| Connective Tissue Disease (Lupus, Scleroderma) | 2% | 0% | 1% |
| Compression Fractures in back | 0% | 0% | 2% |
| Stomach Disease | 2% | 0% | 9% |
| Liver Disease | 2% | 4% | 2% |

# Supplemental Table 2. Frequency of medication use by class and medication names

| **Medication Class** | **Medication Names** | **Total Uses** |
| --- | --- | --- |
| Antidepressant - SSRI | Citalopram, Lexapro, Paroxetine, Prozac | 15 |
| Antidepressant - SNRI | Cymbalta, Venlafaxine | 3 |
| Antidepressant - NDRI | Bupropion | 3 |
| Antidepressant - SARI | Trazodone | 3 |
| Stimulant | Adderall, Amphetamine | 7 |
| Benzodiazepine | Ativan, Diazepam, Lorazepam, Valium | 6 |
| Mood stabilizer | Lamotrigine, Lithium | 3 |
| Antipsychotic - Atypical | Abilify, Quetiapine, Risperdal | 2 |

Note. N=195.

Supplemental Table 3. Self-reported patterns of cannabis use per day vs. days using per month
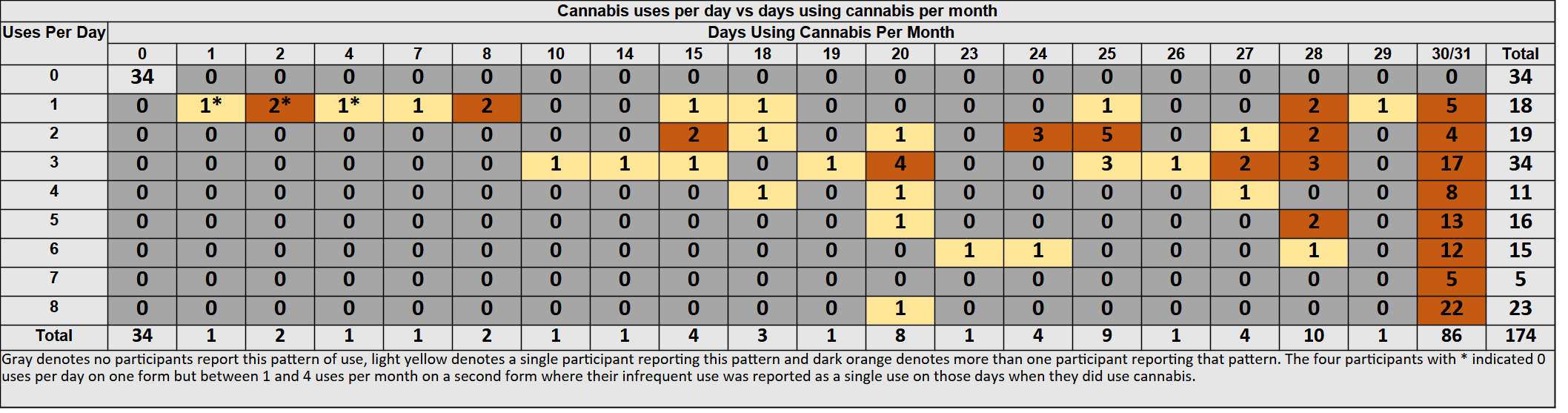


# Supplemental Table 4. Examination of multicollinearity in regression models with Variance Inflation Factor (VIF) values

| **Variable** | **VIF** | **Tolerance** | **HighVIF_5** | **HighVIF_10** |
| --- | --- | --- | --- | --- |
| FreqGroup | 1.031753 | 0.9692245 | FALSE | FALSE |
| age | 1.297083 | 0.7709605 | FALSE | FALSE |
| gender | 1.049270 | 0.9530432 | FALSE | FALSE |
| education_1 | 1.121342 | 0.8917882 | FALSE | FALSE |
| PSQI | 1.215325 | 0.8228254 | FALSE | FALSE |
| any_psychmed | 1.043877 | 0.9579677 | FALSE | FALSE |

Note. N=195. Results show no concerns of multicollinearity.


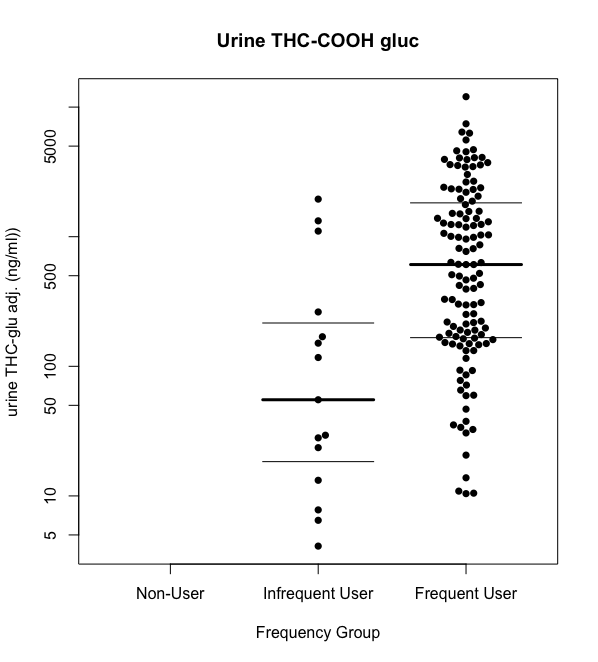


Supplemental Figure 1. Frequent cannabis users have significantly higher urinary THC urinary compared to infrequent cannabis users (1.46 ± 3.09 ng/ml versus 9.58 ± 26.2 ng/ml; *p* = 0.0127). Urine was obtained from the first morning void and levels were adjusted by the plasma/urine creatine measurements. The y-axis is logged and thus subjects with no measurable THC (non-users) are not plotted.
